# Urgent Work for Press and Parliament

**Published:** 1919-08-16

**Authors:** 


### CHOLERA AND SICKNESS.

### URGENT WORK FOR PRESS AND PARLIAMENT.

WE reprint on the following page of THE HOSPITAL to-day an exact copy of a letter as published in the *Times* of August 12. It warns the Government that the nation cannot suffer any repetition of the scandals of Mesopotamia to occur on the North-West Frontier of India. From June last authentic information has reached this country indicating that a lot of sickness was feared there, as the conditions for troops were very bad. Further, the means at hand for dealing with it at the front, including the medical organisation, were bad, the one redeeming feature being an excellent motor ambulance convoy, the men with it being all European officers and men, and therefore reliable. Then, too, the medical *personnel* with the regiments and the field ambulances were bad too, for causes stated by Sir Henry Burdett in his letter to the *Times*.

The supreme question of the moment is, What are the actual conditions of the medical organisation on the North-West Frontier, its equipment, and the quality and number of the R.A.M.C. fully qualified medical staff? Unless the Press and Parliament can immediately bring pressure of the strongest nature to bear upon the Indian authorities, and insist that full and precise details of the actual position of affairs in all respects in regard to the medical organisation shall be presented to Parliament without suppression of any kind before the Houses rise, both Houses will in effect desert our splendid soldiers, who may then, possibly, be left to suffer scandals even greater than those of Mesopotamia. We therefore hope our lay contemporaries, from the *Times* downwards, will combine to compel the Indian authorities immediately and fully to state the actual facts as they exist at the front in India, both in regard to the cholera outbreak, and the whole disease and sickness, to include both the Khyber and Kohat lines. It was rumoured in India, according to the *Morning Post's* Calcutta correspondent of July 17, that the troops were dying like flies from cholera, but the official communiques never used the word cholera or disease, and it was only on July 15 that the public were allowed to know that in the cholera epidemic in June there were 1,663 cases and 566 deaths. Most of the cases and deaths were amongst the followers, but six British officers died and fifteen Europeans of other ranks. Were the six British officers who died doctors? If so, did they include all the European doctors available at the front?

Then in regard to sickness, what the same correspondent describes as a belated message states that the total number of evacuations to hospitals have been: British 2,289, and Indian 5,541. These figures include both the Khyber and Kohat lines. How much these figures conceal, and how much they disclose, there is nothing to show. The people throughout the Empire, especially in this country and India, remembering the Indian Government's record regarding Mesopotamia, relies on Parliament and the Press to cause the India Office plainly to state before the Houses rise, **what is the actual condition of the medical organisation, and equipment, which is known to have been bad in the beginning of June, as described by Sir Henry Burdett, is in fact to-day? Both Parliament and th Press have here an opportunity to free the profession from the risk of a repetition of the scandals of Mesopotamia by securing at once that the Indian Government (if it has not already done so) shall find the money and bring the medical arrangements for our men up to the highest and necessary standard. That standard must entirely supersede the defective medical organisation of the beginning of June, and must provide, without a moment's delay, the most complete and efficient organisation and equipment to render any repetition of the Mesopotamia iniquities impossible, as it was certainly possible two months ago.**

The gravity of the position to-day is demonstrated by the statements received from Calcutta correspondents dated mid-July, an issue of the *Times of India* dated July 17, and the action of the Government in India and at home in suppressing the facts. This unstatesmanlike policy of Mr. Montagu and the Government, and the persistent minimising of the essential gravity of the situation on the North-West Frontier, were dealt with forcibly in leading articles in the *Times* of July 23 and subsequently. This culpable silence and suppression must be resisted by Parliament and the Press, who together can make impossible that it shall longer continue or be tolerated by the people of the British Empire. **It is essential that the exact position in these matters at the present moment shall at once be given to Parliament and promptly made public through the Press.**

